# Cardiovascular risk assessment tools in Asia

**DOI:** 10.1111/jch.14336

**Published:** 2022-01-04

**Authors:** Yuqing Zhang, Huanhuan Miao, Yook‐Chin Chia, Peera Buranakitjaroen, Saulat Siddique, Jinho Shin, Yuda Turana, Sungha Park, Kelvin Tsoi, Chen‐Huan Chen, Hao‐Min Cheng, Yan Li, Huynh Van Minh, Michiaki Nagai, Jennifer Nailes, Jorge Sison, Arieska Ann Soenarta, Guru Prasad Sogunuru, Apichard Sukonthasarn, Jam Chin Tay, Boon Wee Teo, Narsingh Verma, Tzung‐Dau Wang, Satoshi Hoshide, Kazuomi Kario, Jiguang Wang

**Affiliations:** ^1^ Department of Cardiology Fu Wai Hospital Chinese Academy of Medical Sciences and Peking Union Medical College Beijing China; ^2^ Department of Medical Sciences School of Healthcare and Medical Sciences Sunway University Bandar Sunway Selangor Darul Ehsan Malaysia; ^3^ Department of Primary Care Medicine Faculty of Medicine University of Malaya Kuala Lumpur Malaysia; ^4^ Department of Medicine Division of Hypertension Faculty of Medicine Siriraj Hospital Mahidol University Bangkok Thailand; ^5^ Punjab Medical Center Lahore Pakistan; ^6^ Faculty of Cardiology Service Hanyang University Medical Center Seoul Korea; ^7^ School of Medicine and Health Sciences Atma Jaya Catholic University of Indonesia Jakarta Indonesia; ^8^ Division of Cardiology Cardiovascular Hospital, Yonsei Health System Seoul Korea; ^9^ JC School of Public Health and Primary Care The Chinese University of Hong Kong Shatin Hong Kong; ^10^ Institute of Public Health and Community Medicine Research Center National Yang‐Ming University School of Medicine Taipei Taiwan; ^11^ Department of Medicine Division of Cardiology Taipei Veterans General Hospital Taipei Taiwan; ^12^ Faculty of Medicine National Yang‐Ming University School of Medicine Taipei Taiwan; ^13^ Department of Medical Education Center for Evidence‐based Medicine Taipei Veterans General Hospital Taipei Taiwan; ^14^ Centre for Epidemiological Studies and Clinical Trials and Center for Vascular Evaluations Shanghai Key Lab of Hypertension Shanghai Institute of Hypertension Ruijin Hospital Shanghai Jiaotong University School of Medicine Shanghai China; ^15^ Department of Internal Medicine University of Medicine and Pharmacy Hue University Hue City Vietnam; ^16^ Department of Internal Medicine General Medicine and Cardiology Hiroshima City Asa Hospital Hiroshima Japan; ^17^ Department of Preventive and Community Medicine and Research Institute for Health Sciences University of the East Ramon Magsaysay Memorial Medical Center Inc. Quezon City Philippines; ^18^ Department of Medicine Section of Cardiology Medical Center Manila Manila Philippines; ^19^ Department of Cardiology and Vascular Medicine Faculty of Medicine University of Indonesia‐National Cardiovascular Center Harapan Kita Jakarta Indonesia; ^20^ MIOT International Hospital Chennai Tamil Nadu India; ^21^ College of Medical Sciences Kathmandu University Bharatpur Nepal; ^22^ Department of Internal Medicine Cardiology Division Faculty of Medicine Chiang Mai University Chiang Mai Thailand; ^23^ Department of General Medicine Tan Tock Seng Hospital Singapore Singapore; ^24^ Department of Medicine Division of Nephrology, Yong Loo Lin School of Medicine Singapore Singapore; ^25^ Department of Physiology King George's Medical University Lucknow India; ^26^ Department of Internal Medicine, Cardiovascular Center and Division of Cardiology National Taiwan University Hospital Taipei City Taiwan; ^27^ Department of Internal Medicine Division of Hospital Medicine National Taiwan University Hospital Taipei City Taiwan; ^28^ Department of Medicine Division of Cardiovascular Medicine Jichi Medical University School of Medicine Tochigi Japan; ^29^ Department of Hypertension Centre for Epidemiological Studies and Clinical Trials the Shanghai Institute of Hypertension Shanghai Key Laboratory of Hypertension Ruijin Hospital Shanghai Jiaotong University School of Medicine Shanghai China

**Keywords:** Asian patients, cardiovascular disease, hypertension—general, risk assessment

## Abstract

Cardiovascular disease (CVD) is becoming the most important burden to health care systems in most part of the world, especially in Asia. Aiming at identifying high risk individuals and tailoring preventive treatment, many cardiovascular risk assessment tools have been established and most of them were developed in Western countries. However, these cardiovascular risk assessment tools cannot be used interchangeably without recalibration because of the different risk factor profiles (ie, greater absolute burden of hypertension and lower level of total‐cholesterol in Asians and higher prevalence of metabolic disorders in South Asians) and different CVD profiles (higher ratio of stroke/coronary heart disease in Asians) between Western and Asian populations. Original risk models such as Prediction for ASCVD Risk in China (China‐PAR) and Japan Arteriosclerosis Longitudinal Study (JALS) score have been developed and well validated for specific countries, while most of countries/regions in Asia are using established models. Due to higher incidence of stroke in Asians, risk factors like hypertension should weigh more in cardiovascular risk assessment comparing with Western populations, but their actual proportions should be based on CVD profiles in specific countries/regions. The authors encourage the development of new cardiovascular risk assessment tools for Asians, if possible. Still, modifying established models with native epidemiological data of risk factor as well as CVD is acceptable in regions where health care resources are insufficient.

## 1 INTRODUCTION

Cardiovascular disease (CVD), as the leading cause of premature death worldwide, has become the most important burden on health care systems over the past few decades.^1^ It is estimated that about 50% of CVD events occur in Asia where a large proportion of the world population lives. The age‐adjusted mortality of CVD in Asia is also higher,^1^ and this might be attributable to the limited health care systems in many parts of this region due to lower level of development in many parts of the region compared with that of Western developed countries. There are many established risk factors of CVD, that is, age, sex, smoking, hypertension, and diabetes mellitus. Among these risk factors, hypertension is an important component because of its high prevalence and its leading role among preventable causes of premature death in the world.^2^ The population attributable fractions of hypertension for CVD were 25.1% in Australia and New Zealand and 28.6% in East Asia, out‐weighing the separate effects of smoking, high total cholesterol (total‐C) and overweight.^3^ Thus, hypertension control is of great importance for the prevention of further fatal and non‐fatal CVD in this part of the world.

Hypertension is a cardiovascular syndrome and may complicated with other risk factors of CVD. The clustering of these risk factors of CVD tend to interact on target organs and lead to further increase in CV risk. Aiming at identifying individuals that benefit most from intervention and allocating health resources rationally, many risk assessment models and tools for predicting the risk of CVD in the general population as well as in the hypertensive population have been developed.^4^, ^5^, ^6^, ^7^, ^8^, ^9^, ^10^ Currently, many recent hypertension guidelines strongly recommend risk assessment and stratification strategies for hypertensive patients with the purpose of guiding and recommending early intervention for high risk patients and preventing the progression of to severe CVD.^11^, ^12^, ^13^, ^14^, ^15^ These tools are mainly designed for individuals without established CVD and focus on primary prevention. Risk assessment offers a platform for communication between clinicians and patients, improving patients’ awareness of risk, and promoting shared decision‐making, which eventually enhances patients’ adherence to treatment and lead to better clinical practices. It has directive significance for the time to initiate medical treatment, especially statins and aspirin, even though there is lack of consensus surrounding clinically relevant thresholds. Thus, we suggest calibration when using them. Besides, CVD risk assessment tools are increasingly used to estimate risk of individuals and indirectly reflect the effects of several interventions in large randomized controlled trials,^16^ though the applicability is questionable. Furthermore, they have great impact on health care policy making where health inequities and cost‐effectiveness of interventions are often taking into consideration.

However, there is no universal cardiovascular risk assessment tool around the world until most recently. The most widely used tools are mainly developed in Western populations, that is, Framingham CVD risk model,^4^ Systematic COronary Risk Evaluation (SCORE),^5^ and pooled cohort equations (PCEs).^6^ However, taking into consideration of the inequalities of risk factor profiles as well as CVD profiles between Western countries and non‐Western countries, direct application of above‐mentioned tools may result in over or under‐estimation of cardiovascular risk in Asia. Thus, risk assessment tools designed for Asian populations are encouraged, as they may provide more accurate risk prediction.

In this article, we select and evaluate several common risk assessment tools in Western countries, aiming at identifying key attributes of them as well as pointing out their strengths and limitations. More importantly, we also highlight the use of cardiovascular risk assessment tools in Asia by inviting member countries or regions of HOPE‐Asia to provide their tools. Finally, we propose our comments and recommendations for the application of cardiovascular risk assessment tools across a range of resource settings in Asia, with the purpose of proper application of these tools.

## 2 COMMON CARDIOVASCULAR RISK ASSESSMENT TOOLS IN WESTERN COUNTRIES

Currently, the most commonly used risk assessment tools are mainly derived from Western cohorts, with Framingham CVD,^4^ PCE^6^ from American, SCORE^5^ from European, and QRISK3^17^ from United Kingdom. Table 1 shows the basic characteristics of above four cardiovascular risk assessment tools. Framingham coronary heart disease (CHD) risk assessment tool is the first risk assessment tool in the cardiovascular field, which leads the trend of focusing on overall risk of individuals rather than single risk factor.^18^ The subsequent Framingham CVD model adjusted the original risk calculator by incorporating stroke, peripheral artery diseases, and heart failure into its outcomes.^4^ Furthermore, it also presented the definition and metrics of an individual's heart age with the aim of facilitating patients’ understanding of the CVD risk. The risk factors included in the Framingham model are age, sex, smoking, systolic blood pressure (SBP), total‐C, high‐density lipoprotein cholesterol (HDL‐C), hypertensive treatment status, and diabetes.^4^ As the first in the area of cardiovascular risk assessment, this model has been validated in different populations and its modified version has been widely used in many regions, although there are several inherent limitations like the predominantly white Framingham sample (limited representativeness), historically dated populations, and end points that are need to be cautiously explained.^4^, ^19^

**TABLE 1:** Characteristics of currently common cardiovascular risk assessment tools in Western countries

Risk assessment tools	Framingham CVD^4^	Systematic COronary Risk Evaluation (SCORE)^5^	Pooled cohort equations (PCE)^6^	QRISK3^17^
Derivation cohort(s)	Framingham original cohort and Framingham offspring cohort	12 European cohorts in 11 countries	ARIC, CARDIA, CHS, Framingham cohorts	QRESEARCH database
Sample size	8491 participants (4522 women)	205 178 participants (88 080 women)	24 626 participants (11 381 women)	10.56 million participants (5.38 million women)
Age range	30–74	40–65	40–79	25–84
Follow‐up (years)	12	2.7 million person years	At least 12	4.4 (median)
Risk factors	Age; sex; smoking; BP; total‐C; HDL‐C; hypertensive treatment status; diabetes	Age; sex; smoking; SBP; total‐C or total‐C: HDL‐C ratio (low‐risk countries and high‐risk countries versions)	Age; sex; race (white/African American); smoking; SBP; total‐C; HDL‐C; hypertensive treatment status; diabetes	Age; sex; ethnicity; smoking; SBP; total‐C: HDL‐C ratio; BMI; family history; hypertensive treatment status; diabetes; CKD; AF; RA; Townsend deprivation score; a measure of SBP variability; migraine; corticosteroids; SLE; atypical antipsychotics; severe mental illness; erectile dysfunction
End points	10‐year CVD events (CHD, stroke, PAD, or HF)	10‐year fatal CVD events	10‐year hard ASCVD events (non‐fatal MI or CHD death or fatal or non‐fatal stroke)	10‐year CVD events (CHD, ischemic stroke, or TIA)
Tool characteristics	Risk score sheets (tables)	Risk charts	Risk calculators	Risk calculators
Statistical analysis	c‐statistics: 0.763 in men; 0.793 in women χ^2^ statistics: 13.48 in men; 7.79 in women	ROC area: 0.70–0.84 in different European cohorts	c‐statistics: 0.713–0.818 χ^2^ statistics: 4.86–7.25	R^2^: 59.6% in women; 54.8% in men D statistic: 2.48 in women; 2.26 in men Harrell's C statistic: 0.88 in women; 0.86 in men

*Abbreviations*: AF, atrial fibrillation; ARIC, Atherosclerosis Risk in Communities study; ASCVD, arteriosclerotic cardiovascular disease; BMI, body mass index; CARDIA, Coronary Artery Risk Development in Young Adults study; CHD, coronary heart disease; CHS, Cardiovascular Health Study; CKD, chronic kidney disease; CVD, cardiovascular disease; HDL‐C, high‐density lipoprotein cholesterol; HF, heart failure; MI, myocardial infraction; PAD, peripheral artery disease; RA, rheumatoid arthritis; ROC, receiver operating characteristics curve; SBP, systolic blood pressure; SLE, systemic lupus erythematosus; TIA, transient ischemic attack; total‐C, total cholesterol.

SCORE has incorporated traditional risk factors except for diabetes status, with two versions separately for low‐risk countries and high‐risk countries in Europe.^5^ Due to large sample size and resources from multiple countries’ cohorts, it has great representativeness of European populations and acquires internal validation among these countries. However, non‐fatal CVDs like non‐fatal myocardial infraction (MI) also impose severe burden on individuals’ health as well as social medical security system, are not evaluated in the SCORE tool and hence cannot be predicted through the SCORE tool. Furthermore, it may underestimate individuals’ cardiovascular risk in countries with a very‐high background risk and hence whether it is applicable in other populations remains uncertain.

PCE can be considered as an upgrade of the Framingham model where it includes Atherosclerosis Risk in Communities study (ARIC), Coronary Artery Risk Development in Young Adults study (CARDIA), and Cardiovascular Health Study (CHS) cohorts besides the Framingham cohorts. PCE shares the same risk factors as the original Framingham model, but now with inclusion of different ethnic groups making it more applicable in both Whites and African Americans.^6^ Comparing with the original Framingham CHD model, PCE has a broader range of applicable populations and more comprehensive end points. Due to its main purpose of instructing lipid‐lowering therapies, it mainly evaluates and predicts the risk of arteriosclerotic CVD (ASCVD), which is more common in Western countries. And when it comes to inform antihypertensive treatment, extra attention should be put on the risk of hemorrhagic stroke that accounts for a considerable proportion in CVD, especially among non‐Western countries.^1^

Recently, the Million Hearts Longitudinal ASCVD risk assessment tool was developed on the basis of PCE tool. Notably, this tool could provide not only baseline 10‐year ASCVD risk estimates, but also updated 10‐year ASCVD risk estimates after implementing cardiovascular preventive strategies (aspirin therapy, blood pressure management, cholesterol management, and smoking cessation), serving as important supplement to risk estimates for individuals at follow‐up.^20^ However, its applicable populations are basically identical to PCE, limiting its generalization to other populations. Still, further studies are needed to validate its precision and applicability.

QRISK3 is designed to predict cardiovascular risk in the United Kingdom, which is based on the national QRESEARCH database and overall data resources from 10.56 million participants.^17^ Unlike the other tools, QRISK3 contains a few non‐traditional risk factors, like ethnicity, body mass index (BMI), social deprivation, family history, a measure of SBP variability, special medicine (like corticosteroids and atypical antipsychotics), and specific diseases associated with high cardiovascular risk (like chronic kidney disease, systemic lupus erythematosus, etc).^17^ Including the additional above‐mentioned risk factors may enhance the accuracy and precision of individuals’ risk assessment in United Kingdom, as it promotes attention to social issues like health inequalities and may propel reallocation of health resources appropriately.

## 3 CURRENT STATUS OF CARDIOVASCULAR RISK ASSESSMENT TOOLS IN ASIA

Because more than half of CVD events worldwide occur in Asia, prevention through targeting the most vulnerable individuals is the priority to reduce the CVD burden in this region. Although there are many cardiovascular risk assessment tools available, only a few of them are originally derived from Asian populations. There are many differences in the risk factor and CVD profiles between the Asian and Western populations. Many countries and regions in the East Asia have greater absolute burden of hypertension, but with lower rates of awareness, treatment, and control.^2^ Epidemiologic studies presented that population attributable fraction for CVD related to hypertension were around 25–30% in Asia‐Pacific region, indicating that more attention should be paid to hypertension while predicting individuals’ cardiovascular risk.^3^, ^21^ While the SBP and total‐C were reported to be lower in Asian cohorts, comparing with the Framingham cohort, the rate of smoking in men was higher.^22^ Diabetes is more prevalent among South Asians, along with lower levels of HDL‐C.^23^ Furthermore, the proportion of subtypes of CVD also differs. As evidenced by a meta‐analysis, fatal stroke/CHD ratios were 1.5:1 in a long‐term period (over 10 years) and 2:1 in a short‐term period in Asians, while the Western population has the opposite ratios (long‐term 1:3 vs. short‐term 1:4 in men and 1:2 in women).^24^ Given the high prevalence of metabolic diseases especially diabetes mellitus in South Asians, the prevalence of stroke is still higher than CHD in this area, which might be attributable to uncontrolled higher DBP and relatively lower total‐C and LDL‐C than Western populations.^25^

Table 2 shows the characteristics of cardiovascular risk assessment tools in several Asian regions. Prediction for ASCVD Risk in China (China‐PAR), was derived from two contemporary Chinese cohorts and validated in two independent Chinese cohorts, is an effective risk assessment tool in China, aiming at identifying high risk individuals in terms of the 10‐year ASCVD risk.^9^ The end point events were defined as non‐fatal acute MI or CHD death or non‐fatal or fatal stroke, similar to PCE.^9^ As for risk factors, other than traditional risk factors, four non‐traditional risk factors (waist circumference [WC], geographic region, urbanization, and family history) were added to the equation for men, and two of them (WC and geographic region) were added to the equation for women, for their relative integrated discrimination improvement indices ≥6% (predefined inclusion criterion).^9^ One study showed it has great ability of discrimination (c‐statistic: 0.811 for women and 0.794 for men) and calibration (χ^2^ statistics: 12.8 for women and 13.1 for men), with a better performance than PCE in predicting 10‐year ASCVD risk among Chinese populations.^9^ WC, as an indicator of visceral fat, is closely associated with ASCVD risk but whether there are confounding factors remains unknown. Geographic region and urbanization variables focusing on the gap of incidence of ASCVD both between Northern and Southern China and between urban and rural areas can enhance the precision capability of the tool. On the other hand, it makes the tool more complicated and harder to generalize to other populations.

**TABLE 2:** Characteristics of currently common cardiovascular risk assessment tools in Asian region

Country/region	Risk assessment tools	Original/established	Derivation cohort(s)	Age range	Risk factors
China^9^	China‐PAR	Original	InterASIA and China MUCA (1998)	N/A	Age; sex; smoking; SBP; total‐C; HDL‐C; hypertensive treatment status; diabetes; WC; geographic region; urbanization (only for men); family history of ASCVD (only for men)
Japan^8^	JALS score	Original	JALS cohorts	40–89	Age; sex; smoking; BP; non‐HDL‐C; HDL‐C; hypertensive treatment status; diabetes; BMI; eGFR; AF (AF model and non‐AF model)
Malaysia^26^	Framingham CVD^4^	Established	Framingham original cohort and Framingham offspring cohort	30–74	Age; sex; smoking; SBP; total‐C; HDL‐C; hypertensive treatment status; diabetes
Indonesia^10^	WHO/ISH chart	Established	N/A	≥40	Age; sex; smoking; SBP; total‐C; diabetes
Vietnam^27^	Framingham CVD^4^	Established	Framingham original cohort and Framingham offspring cohort	30–74	Age; sex; smoking; SBP; total‐C; HDL‐C; hypertensive treatment status; diabetes
Taiwan^28^, ^29^	A point‐based prediction model	Original	CCCC original cohort	≥35	Clinical model: Age; sex; BMI; SBP; smoking Total‐C‐based model: Age; sex; BMI; SBP; total‐C; HDL‐C LDL‐C‐based model: Age; sex; BMI; SBP; LDL‐C; HDL‐C
	Framingham CHD^18^	Established	Framingham original cohort/TwSHHH	35–70	Age; sex; smoking; SBP; total‐C; HDL‐C; diabetes
Singapore^30^	Framingham ATPIII^31^	Established	Framingham cohort	20–79	Age; sex; smoking; SBP; total‐C; HDL‐C; hypertensive treatment status

Country/region	End points	Statistic characteristics	External validation	Recommended by native guideline (yes/no)	Comments/developments
China^9^	10‐year ASCVD events (non‐fatal acute MI or CHD death or fatal or non‐fatal stroke)	c‐statistics: 0.794 in men; 0.811 in women χ^2^ statistics: 13.1 in men; 12.8 in women	Yes	No	
Japan^8^	5‐/10‐year stroke/acute MI/composite outcome of stroke and acute MI/all cardiovascular death events	ROC area: (model of all cardiovascular death prediction) 0.828 in non‐AF model 0.832 in AF model	No	Yes	
Malaysia^26^	10‐year CVD events (CHD, stroke, PAD, or HF)	ROC area: 0.63 χ^2^ statistics: 3.25	Yes	No	CKD is advised in risk prediction for medium‐risk patients.^32^
Indonesia^10^	10‐year fatal or non‐fatal major cardiovascular events (myocardial infarction or stroke)	N/A	N/A	No	
Vietnam^27^	10‐year CVD events (CHD, stroke, PAD, or HF)	N/A	Yes	N/A	Modification and calibration of an existing score for the Vietnamese population.
Taiwan^28^, ^29^	10‐year incident CHD events (fatal and nonfatal MI and cases undertaking PCI or CABG)	ROC area: 0.73–0.78; IDI: 0.2%, *p* = .11 NRI: 8.2%, *p* = .11	Yes	No	Models as well as point systems were developed.
	10‐year CHD events	χ^2^ statistics: 5.668 in men; 389.086 in women	Yes	Yes	The adjustment factors were submitted to the International Society of Lipids and Atherosclerosis.
Singapore^30^	10‐year hard CHD events (MI and cardiovascular death)	N/A	Yes	Yes	The tool was modified and adjusted for three ethnic groups in Singapore (Chinese, Malay, and Indian).

*Abbreviations*: AF, atrial fibrillation; ASCVD, arteriosclerotic cardiovascular disease; ATP III, Third Report of the Adult Treatment Panel; BMI, body mass index; CCCC, Chin‐San Community Cardiovascular Cohort; CHD, coronary heart disease; China MUCA, China Multi‐Center Collaborative Study of Cardiovascular Epidemiology; China‐PAR, Prediction for ASCVD Risk in China; CKD, chronic kidney disease; CVD, cardiovascular disease; HDL‐C, high‐density lipoprotein cholesterol; HF, heart failure; IDI, integrated discrimination improvement; InterASIA, International Collaborative Study of Cardiovascular Disease in Asia; JALS, Japan Arteriosclerosis Longitudinal Study; LDL‐C, low‐density lipoprotein cholesterol; MI, myocardial infraction; NRI, net reclassification improvement; N/A, data unavailable; PAD, peripheral artery disease; ROC, receiver operating characteristics curve; SBP, systolic blood pressure; total‐C, total cholesterol; TwSHHH, Taiwanese Survey on Hypertension, Hyperglycemia, and Hyperlipidemia; WC, waist circumference; WHO/ISH, World Health Organization/International Society of Hypertension.

Japan Arteriosclerosis Longitudinal Study (JALS) score is designed to estimate 5‐ and 10‐year absolute risk for stroke, acute MI, composite outcome of stroke and acute MI, and CVD death, respectively, based on a large cohort study in Japan with a median follow‐up of 6.9 years.^8^ In addition to traditional risk factors, the tool has incorporated BMI and estimated glomerular filtration ratio into the score for the risk of CVD death and established with/without AF models within each score.^8^ However, different from previous findings, the study showed that lower BMI, instead of overweight and obesity, is the risk factor for CVD death. Interestingly, the study first reported that among individuals with Grade Ⅱ or III hypertension, the cardiovascular risk of treated hypertension is lower than untreated hypertension. Although the reason is unclear, it challenges conventional perspective that patients with treated hypertension are at a higher risk compared with patients with untreated and similar level of BP.^26^ Further studies are needed to explain this discrepancy. From another perspective, it does highlight the importance of predicting updated risk after intervention with new tools (eg, Million Hearts Longitudinal ASCVD risk assessment tool) rather than initial risk assessment tools. Given the median follow‐up of less than 10 years, the prediction of 10‐year risk in this tool should be tested in future studies. The Hisayama study^27^ is also recommended by the Japanese hypertension guideline 2019^14^ for risk assessment, but its sample size is much smaller. The estimated vascular age provided by Hisayama study presents an intuitive form of individuals’ risk, which could facilitate patients’ perception of cardiovascular risk and motivate their adherence to treatment.

World Health Organization/International Society of Hypertension (WHO/ISH) chart is also being used, especially in many countries and regions in Southeast Asia,^10^ This is a risk prediction tool particular for low and middle‐income countries (LMICs) where health care resources and national epidemiological data are insufficient. The prediction tool uses major risk factors (age, sex, smoking, SBP, total‐C, and diabetes) to assess 10‐year risk of acute MI and stroke. Given that total‐C might be unavailable in some settings, the tool has developed two versions: total‐C included/total‐C excluded, and the two versions were shown to have close correlation in risk prediction.^28^ A study has shown that WHO/ISH chart performed in a similar manner to the Framingham risk models and SCORE in risk prediction among South Africans.^28^ Additionally, WHO/ISH chart is an easy‐to‐use tool that facilitates its generalization to considerable LMICs. WHO package of essential noncommunicable disease interventions (WHO PEN) is an initiative project aiming at prioritize cost‐effective tools and interventions to low‐resource settings, in which WHO/ISH chart was strongly recommended to identify high‐risk groups who would benefit most from interventions.^29^ In order to improve precision of the tool, national epidemiological data can be considered to recalibrate and modify original chart when they are applied in certain countries. Although there are established risk assessment tools for Asian populations by Barzi and colleagues,^22^ major variables like HDL‐C and diabetes were ignored in this tool, limiting its accuracy and applicability. Comparing with establishing large cohorts with long periods of follow‐up to derive new risk assessment tools, investigating and monitoring risk factors as well as the morbidity and mortality of CVD nationwide to modify WHO/ISH chart might be a priority for many LMICs. Overall, this tool can be adopted by most LMICs in Asia where original cardiovascular risk assessment tools are unavailable.

Based on available evidence, there are also marked variations in risk profiles as well as CVD profiles among different regions in Asia. East Asians have higher prevalence of hypertension, relatively lower total‐C, which might provide an explanation for higher ratio of stroke: CHD as well as hemorrhagic: ischemic stroke in this area. Unlike East Asians, South Asians suffers from higher rates of dyslipidemia (ie, lower level of HDL‐C, elevated lipoprotein (a)), insulin resistance, and diabetes, whereas lower rates of hypertension, obesity, and hypercholesterolemia. As a result, the incidence and mortality of CHD are much higher and the occurrence of CHD is much earlier in South Asians than the other area.^30^ Therefore, CVD risk prediction should be made according to specific national conditions.

Among these risk assessment tools used in different Asia regions (Table 2), original tools are still the minority, while Framingham models are commonly used in many countries/regions with or without recalibration. Besides, WHO/ISH chart is also being used. However, it is reported that Framingham models might overestimate CVD risk in Asians due to much higher blood pressure, TC, and CV events in Framingham cohorts.^31^ Additionally, WHO/ISH chart was reported to underestimate CVD risk in several Asian regions.^32^

As for the desirable attributes of CVD risk assessment tools, they depend on socio‐economic conditions and medical resources. For high‐resource settings, electronic scoring systems are always available and it is convenient to apply them. In contrast, for low‐resource settings, risk charts might be more appropriate for them for their simplicity and clearness.

In summary, we encourage the development of novel CVD risk assessment tools in high resource settings, and recommend recalibration of established models in LMICs. Importantly, the ratio of stroke: CHD as well as ischemic: hemorrhagic stroke should be taken into consideration when selecting and modifying CVD risk assessment tools. Furthermore, we suggest considering different CVD risk assessment tools in regard to different clinical decision to benefit the most, and different versions of the same score for different clinical decisions are potential choices.

While developing original tools, some non‐traditional variables could be incorporated if meaningful. Statistical incremental value might be imperative, which means that the novel risk factors should improve prediction over previous risk assessment tools. There are many statistical performance measures, including calibration (eg, calibration plot, ratio of observed to expected events), discrimination (eg, c‐statistic and area under the curve), and reclassification (eg, net reclassification index, integrated discrimination improvement).^33^ However, there is a robust debate about which measures are more useful to evaluate novel risk factors. Besides, whether they could instruct clinical practices and promote clinical outcomes should also be taken into consideration.^34^ Furthermore, from the prospective of public health, cost‐effectiveness and health equalities are of great importance. Some social risk factors like social‐economic status are possible candidates. However, their ascertainment is critically based on methods and it is hard to standardize them. Besides, with the rapid development of society, SES parameters also change quickly and it is hard to identify current SES based on data from 10 years ago.

Unfortunately, the utilization of CVD risk assessment tools is relatively low even in high resource settings. Hitherto, no RCT has demonstrated its clinical benefit. But it should be highlighted that risk assessment tools per se cannot promote patients’ outcomes unless being properly used in clinical practices and we urge the widely use and test of them.

## 4 CONCLUSIONS

Given that risk factors and CVD profiles are of some different between Western and Asian populations, cardiovascular risk assessment tools cannot be used interchangeably without recalibration. Due to higher incidence of stroke in Asians, risk factors like hypertension should weigh more in cardiovascular risk assessment comparing with Western populations, but their actual proportions should be based on CVD profiles in specific countries/regions. The ratio of stroke: CHD as well as hemorrhagic: ischemic stroke should be taken into consideration when developing and modifying CVD risk assessment tools.

Original and native risk models for Asian populations are still limited. While using current local risk assessment tools in the specific regions in Asia that need to be improved in future studies, we encourage the establishment of current, large, representative cohorts to develop new CVD risk assessment tools and taking into account some non‐traditional risk variables. On the other hand, modifying and recalibrating established models like WHO/ISH chart and Framingham CVD model might also be solutions in many LMICs where health care resources are insufficient.

## CONFLICT OF INTEREST

Y Zhang has received research grants from Bayer, Novartis, and Shuanghe; and lecture fees from Bayer, Daiichi Sankyo, Novartis, Pfizer, Sanofi, Servier, and Takeda. J Shin has received honoraria and sponsorship to attend seminars from Daiichi Sankyo, Takeda, Menarini, MSD, Bristol‐Myers Squibb, and Sanofi. K Kario  received research grant from A &D Co.; Omron Healthcare Co.; Roche Diagnostics KK; MSD KK; Astellas Pharma Inc; Otsuka Holdings Co.; Otsuka Pharmaceutical Co.; Sanofi KK; Shionogi & Co.; Sanwa Kagaku Kenkyusho Co.; Daiichi SankyoCo.; Sumitomo Dainippon Pharma Co.; Takeda Pharmaceutical Co.; Mitsubishi Tanabe Pharma Co.; Teijin Pharma; Boehringer Ingelheim Japan Inc; Pfizer Japan Inc; and Fukuda Denshi Co. S Siddique has received honoraria from Bayer, GlaxoSmithKline, Pfizer, ICI, and Servier; and travel, accommodation, and conference registration support from Atco Pharmaceutical, Highnoon Laboratories, Horizon Pharma, ICI, and Pfizer. Y‐C Chia has received honoraria and sponsorship to attend conferences and CME seminars from Abbott, Bayer, Boehringer Ingelheim, GlaxoSmithKline, Menarini, Merck Sharp & Dohme, Novartis, Orient Europharma, Pfizer, and Sanofi; and a research grant from Pfizer. C‐H Chen has received honoraria as a member of a speaker's bureau for Pfizer. J Sison has received honoraria from Pfizer, AstraZeneca, Boehringer Ingelheim, and Novartis. GP Sogunuru has received a research grant related to hypertension monitoring and treatment from Pfizer. JC Tay has received advisory board and consultant honoraria from Pfizer. JG Wang has received research grants from Bayer, Pfizer, and Phillips; and lecture and consulting fees from Bayer, Daiichi‐Sankyo, Merck Sharp & Dohme, Pfizer, Sanofi, and Servier. All other authors report no potential conflicts of interest in relation to this article.

## AUTHOR CONTRIBUTIONS

This study was done on behalf of the Hypertension Cardiovascular Outcome Prevention and Evidence (HOPE) Asia Network. Yuqing Zhang and Huanhuan Miao drafted the manuscript. Yook‐Chin Chia, Peera Buranakitjaroen, Saulat Siddique, Jinho Shin, Yuda Turana, Sungha Park, Kelvin Tsoi, Chen‐Huan Chen, Hao‐Min Cheng, Yan Li, Huynh Van Minh, Michiaki Nagai, Jennifer Nailes, Jorge Sison, Arieska Ann Soenarta, Guru Prasad Sogunuru, Apichard Sukonthasarn, Jam Chin Tay, Boon Wee Teo, Narsingh Verma, Tzung‐Dau Wang, Satoshi Hoshide, Kazuomi Kario, and Jiguang Wang provided their comments and revisions of the document.
